# Avoided Hospitalization Criteria: Validating the Impact of a Community-Based Palliative Care Program

**DOI:** 10.1089/pmr.2020.0077

**Published:** 2020-10-27

**Authors:** Gobi Paramanandam, Barbara E. Volk-Craft, Rachel Mayer Brueckner, Theresa M. O'Sullivan, Erin Waters

**Affiliations:** Palliative Care Program, Hospice of the Valley, Phoenix, Arizona, USA.

**Keywords:** acute care utilization, avoided hospitalization, home-based palliative care

## Abstract

***Objective:*** This report describes the experiences of a community-based palliative care (CBPC) program's efforts to understand the patterns of hospital utilization, specifically utilization reduction experienced by admitted patients. Efforts to quantify and describe an avoided hospitalization and opportunities to use these data to strengthen partnerships with local insurance payers to assure sustainability of the CBPC will be discussed.

***Background:*** Patients with serious chronic illness experience emergency room care and hospitalizations with increasing frequency as their health deteriorates. CBPC programs are well positioned to decrease hospital utilization by early involvement and improved care management.

***Methods:*** Arizona Palliative Home Care (AZPHC) program is a free standing CBPC in Maricopa County, Arizona, serving 3300 patients annually. An interdisciplinary team was formed within the CBPC to facilitate the identification of avoided hospital events and communicate these data to community partners in an effective and consistent manner. The processes developed by this team are described.

***Results:*** AZPHC has enhanced its hospitalization avoidance strategies by communicating the rate of hospitalization avoidance events in a consistent and strategic manner. Providing instances of avoided hospitalizations with accompanying patient narratives to payers has enabled AZPHC to demonstrate the impact the CBPC has on improving quality of care and reducing overall costs.

***Discussion:*** CBPC programs require payment for sustainability; therefore, partnerships with local insurance payers are essential. Presenting data that validate the impact of a program from a clinical and financial perspective will advance the growth of payer–CBPC provider relationships and secure a future for funded CBPC programs.

## Introduction

Over 12 million American adults are living with a serious illness, such as cancer, heart disease, kidney disease, or dementia.^1,2^ In Arizona, these chronic diseases are responsible for 7 out of 10 deaths, with the occurrence of multiple concurrent conditions contributing to a higher mortality rate.^3,4^ Patients with serious chronic illness utilize emergency room care and inpatient hospital settings with increasing frequency as their conditions progress and baseline health deteriorates.^5,6^

Community-based palliative care (CBPC) programs are well positioned to reduce hospital utilization by providing earlier interventions and improving care management.^7–14^ In the absence of federally mandated reimbursement, an interdisciplinary CBPC program can establish a direct partnership with payers and demonstrate the positive fiscal impact of palliative care services.^15–19^ For insurance payers, decreased hospital utilization results in avoided or reduced expenditures as less expensive community-based services are provided instead.^20–23^

## Setting

Arizona Palliative Home Care (AZPHC) program is a free standing CBPB serving 3300 patients annually with an average daily census of 570. AZPHC offers coordination of care to late-stage chronically ill patients struggling with daily living and disease management. Patients can receive this palliative care concurrently with disease-directed treatments and the AZPHC team of medical providers, registered nurses, social workers, and support staff work collaboratively with each patient's physicians. Key program elements include home visits by a professional nursing/social work case manager, 24/7 registered nurse telehealth support, education about disease management and medications, assistance with advance directives, posthospital support focusing on medication reconciliation and scheduling follow-up care, and volunteer companions for caregiver respite. Initially supported by grants and charitable donations, AZPHC has successfully become a revenue-generating business line for its parent organization, Hospice of the Valley.

## Intervention

### Identifying avoided hospitalizations

AZPHC produces clinical and financial data that have substantiated the value of CBPC for our business partners. Hospital utilization is tracked through the health information exchange (HIE) and CBPC program's electronic medical record (EMR). An emergency room or hospital admission is an *event* that is easily measured.

An event that does not occur—an *avoided hospitalization*—is more challenging to document, although the cost savings and impact on patient well-being are undeniable. Avoided hospitalizations, including emergency department visits, observation days, and inpatient stays, can be used to validate the effectiveness of a CBPC program. With increasing fiscal pressure on insurers,^24–30^ the AZPHC team identified a need to enhance our hospital avoidance strategies and communicate our *rate of avoided hospitalizations* to those payers in a precise, consistent, and strategic manner.

### Developing the avoided hospitalization criteria

AZPHC leadership soon understood that hospitalization reduction was critical for the CBPC program to succeed. Leadership ensured every staff member understood this priority and that it was imbedded as part of the program's organizational culture. As part of this cultural integration, AZPHC created a hospital avoided team with membership of program leaders, medical providers, field clinicians, and support staff. This team did a literature review exploring evidence-based predictors of hospitalizations,^31^ reviewed case studies of hospital events for common themes, and created a list of scenarios based on their clinical experiences. The results of this collaboration became AZPHC's avoided hospital event criteria (Table 1).

**Table 1.** Avoided Hospital Event Criteria

Patient/family/caregiver specifically reference 911 or “going to the hospital.” Patients and families will often report that if it was not for AZPHC interventions, they would have gone to the emergency room. Caregivers especially exhibit anxiety over symptoms and need the prompt guidance and interventions that CBPC programs provide with a 24/7 response.
Patient reporting symptoms that previously required a hospitalization. Occurring frequently in cancer patients with uncontrolled symptoms or patients who are experiencing exacerbations of COPD or CHF, education and intervention in the home setting can be very effective in reducing a hospital readmission, particularly the 30-day post-hospitalization.
Nonscheduled visit is made during business or after hours. A 24/7 urgent response provides immediate telephonic nurse support and skilled nursing visits on weekends and weeknights when a patient's only other option for immediate intervention would be an emergency room visit.
Transition to hospice care from home without terminal hospital event. Many inpatient palliative care teams do an excellent job with nuanced goals of care conversations and subsequently transition patients to hospice care. CBPC programs have the ability to have these conversations over time in the comfort of the home setting. A final and costly hospitalization can be avoided through direct hospice transitions facilitated in the home environment.
Provider changes plan of care/medications due to AZPHC intervention. Patients may be unable to get an urgent appointment with their primary care provider or specialist, or are unable to leave their home due to mobility issues. The AZPHC nurse may contact the AZPHC medical provider while in a home visit due to worsening symptoms. Mobile X-rays, laboratories, and medications can be ordered and symptoms can be managed in the home setting, similar to a mobile urgent care. With patient education and subsequent follow-up with their primary care and specialist providers, a hospitalization can be avoided.
Social work interventions provided to patients who have demonstrated past hospital utilization due to social determinants of health. The CBPC team, with the advantage of entering patient homes, understands that a patient's environment can be the greatest indicator of their health. AZPHC has found that addressing and managing social determinants of health can often lead to decreased hospitalization. Sharing patient-specific examples and narratives with payers has facilitated a fuller understanding of the social impacts on health. This enhanced appreciation of the patient experience has been a very powerful tool during contract negotiations with payers.

AZPHC, Arizona Palliative Home Care; CBPC, community-based palliative care; CHF, chronic heart failure; COPD, chronic obstructive pulmonary disease.

### Implementing the avoided hospitalization process

Initially, AZPHC relied on clinicians (nurses, social workers, and medical providers) to flag potential avoided events for further review. Despite continued education and reinforcement on the importance of this process, the full count of avoided hospital events was not routinely captured. When asked, staff stated that they were unsure whether their intervention constituted an avoided hospitalization and, in their uncertainty, did not mark the patient record for further review. In response, leadership modified the clinical notes in the EMR to add the question: was a hospitalization avoided during your visit? Responses are “yes,” “no,” or “maybe.” A report was created to identify records containing “yes” or “maybe” for further review. Urgent calls to our telehealth nurse, unscheduled home visits to manage acute symptoms, and calls to our after hour triage team were identified on the report. Patient transitions to hospice that occurred in the home without return to the hospital setting were subsequently flagged for further review.

The flagged intervention is followed for one week and a lack of hospitalization is confirmed through review of the HIE records. A chart review by the AZPHC medical director or AZPHC telehealth RN is completed to create a narrative that describes the patient experience, intervention, and clinical outcome.

### Including payers in development of a reporting tool

A report of hospitalizations and avoided hospitalizations, listing hospital event details and patient experience narratives, was shared with each health insurance payer on a monthly basis (Table 2). The AZPHC clinical leadership met together with payer representatives at regular intervals to address recurrent issues or barriers that impact the frequency of hospitalizations. This practice established a relationship of collaboration, transparency, and trust. Including a list of *avoided hospitalizations* further demonstrated the impact of the CBPC program on both improving quality and cost outcomes.

**Table 2.** Hospitalization and Avoided Events Report

Event Date	Event Type	Narrative
May 2, 2020	Hospitalization avoided	Mr. X has a history of prostate cancer. Spouse calls AZPHC telehealth RN to share update from oncologist that treatment has not been effective. RN reviews prognosis and goals of care. Spouse states that they will be remaining in Arizona and requests hospice evaluation. AZPHC team facilitates in-home transition to hospice care without return to hospital setting.
May 5, 2020	Hospitalization avoided	Ms. X has a history of pulmonary fibrosis, atrial fibrillation, CKD, and chronic pain syndrome. AZPHC RN home assessment finds patient almost out of routine pain medicines. Patient unclear who is prescribing or managing pain. Phone consult with pain clinic finds patient's provider has left practice and she has no open referral. Phone consult with PCP to review pain management options and PCP agrees to follow. Patient scheduled for f/u visit to PCP to establish pain Rx. Patient in agreement with plan and grateful for proactive management of medicines.
May 8, 2020	Hospitalization avoided	Ms. X has a history of CHF and CKD 3. She calls AZPHC triage RN to report blood in urine. After hours RN home visit finds patient afebrile without acute distress. Patient reports appearance of blood in urine today with burning with voiding. Reports history of UTIs. Phone consult with PCP and received order for urinalysis culture and oral antibiotic. RN reminds patient regarding importance of hydration and hygiene. Patient in agreement with plan and declines need for ER at this time.
May 13, 2020	Hospitalization avoided	Ms. X has a history of COPD, CHF, and CKD3. Daughter calls AZPHC triage RN to report swelling and pain in elbow. After hours RN home assessment finds patient without report of trauma. Golf ball-sized soft swelling on elbow, painful to touch, red/warm. Afebrile with clear breath sounds and minimal lower extremity edema. Phone consult with PCP on call. Oral pain medicine, immobilizing sling, elevation ordered. Portable X-ray reveals bursitis. PCP home visit scheduled for next business day. Patient and daughter agree with plan and deny need for ER at this time.
May 18, 2020	Hospitalization avoided	Ms. X has a history of dementia and HTN. AZPHC RN assessment finds patient with symptoms of UTI (frequency and odorous urine). Patient reports poor oral intake/hydration. Phone consult with AZPHC provider and new orders for oral antibiotic. Detailed education to patient and caregiver re medicine use and dosing. Reinforced importance of hydration and good hygiene. Patient and caregiver agree with plan and decline need for ER.
May 26, 2020	Hospitalization avoided	Mr. X has a history of CHF, CVA, CAD, and HTN. He calls AZPHC team to report that his neighbor who previously assisted with household chores and food preparation was now not available and he was concerned re access to food. AZPHC social worker contacts ALTCS case manager to verify patient approved for 20 hours caregiving per week and schedules support. HOV volunteer agrees to run errands/grocery shopping for patient. Patient in agreement with plan and pleased to avoid return to inpatient care due to lack of resources.

Identifiable patient data, including patient name, have been removed.

CAD, coronary artery disease; CKD, chronic kidney disease; CVA, cerebral vascular accident; ER, emergency room; HTN, hyptertension; PCP, primary care physician; UTI, urinary tract infection.

## Results

Hospice organizations looking to diversify their service lines and hospitals interested in starting CBPC programs may be interested in concrete examples of decreased utilization to assist in business planning and proposals for program funding. This article is a description of the process that the AZPHC program explored to articulate and share one aspect of utilization, an *avoided hospitalization*, with community partners. Using this process of collaboration, data sharing and most importantly, telling the patient's story, AZPHC has successfully engaged with several health plans and a major hospital system for reimbursed palliative care services.

The process by which the AZPHC program measured impacts on other outcomes of interest, such as high-cost hospital events and emergency department visits, is further described in a prior publication.^32^ The corresponding author of this article welcomes further inquiry from interested colleagues.

## Conclusion

Understanding the patterns of hospital utilization, and specifically utilization reduction, is vital for any CBPC program. CBPC programs require payment for sustainability; therefore, partnerships with local insurance payers are essential. CBPC programs have the potential to greatly improve the quality of care for chronically ill populations and reduce costs for our health care system. Presenting data that validate the clinical and financial impacts will advance the growth of payer–CBPC provider relationships.

## Abbreviations Used

AZPHC: Arizona Palliative Home Care
CBPC: community-based palliative care
CHF: chronic heart failure
CKD: chronic kidney disease
COPD: chronic obstructive pulmonary disease
EMR: electronic medical record
ER: emergency room
HIE: health information exchange
HTN: hypertension
PCP: primary care physician
UTI: urinary tract infection
